# Exploration of programmed cell death-associated characteristics and immune infiltration in neonatal sepsis: new insights from bioinformatics analysis and machine learning

**DOI:** 10.1186/s12887-024-04555-y

**Published:** 2024-01-20

**Authors:** Yun Hang, Huanxia Qu, Juanzhi Yang, Zhang Li, Shiqi Ma, Chenlu Tang, Chuyan Wu, Yunlei Bao, Feng Jiang, Jin Shu

**Affiliations:** 1 Department of Pediatrics, The Fourth Affiliated Hospital of Jiangsu University, Zhenjiang, 212001 China; 2 Department of Blood Transfusion, Zhenjiang First People’s Hospital, Zhenjiang, China; 3 Department of Rehabilitation Medicine, The First Affiliated Hospital of Nanjing Medical University, Nanjing, China; 4 Department of Neonatology, Obstetrics and Gynecology Hospital of Fudan University, Shanghai, 200011 China

**Keywords:** Neonatal sepsis, Machine learning, Diagnosis, Programmed cell death, Immune infiltration

## Abstract

**Background:**

Neonatal sepsis, a perilous medical situation, is typified by the malfunction of organs and serves as the primary reason for neonatal mortality. Nevertheless, the mechanisms underlying newborn sepsis remain ambiguous. Programmed cell death (PCD) has a connection with numerous infectious illnesses and holds a significant function in newborn sepsis, potentially serving as a marker for diagnosing the condition.

**Methods:**

From the GEO public repository, we selected two groups, which we referred to as the training and validation sets, for our analysis of neonatal sepsis. We obtained PCD-related genes from 12 different patterns, including databases and published literature. We first obtained differential expressed genes (DEGs) for neonatal sepsis and controls. Three advanced machine learning techniques, namely LASSO, SVM-RFE, and RF, were employed to identify potential genes connected to PCD. To further validate the results, PPI networks were constructed, artificial neural networks and consensus clustering were used. Subsequently, a neonatal sepsis diagnostic prediction model was developed and evaluated. We conducted an analysis of immune cell infiltration to examine immune cell dysregulation in neonatal sepsis, and we established a ceRNA network based on the identified marker genes.

**Results:**

Within the context of neonatal sepsis, a total of 49 genes exhibited an intersection between the differentially expressed genes (DEGs) and those associated with programmed cell death (PCD). Utilizing three distinct machine learning techniques, six genes were identified as common to both DEGs and PCD-associated genes. A diagnostic model was subsequently constructed by integrating differential expression profiles, and subsequently validated by conducting artificial neural networks and consensus clustering. Receiver operating characteristic (ROC) curves were employed to assess the diagnostic merit of the model, which yielded promising results. The immune infiltration analysis revealed notable disparities in patients diagnosed with neonatal sepsis. Furthermore, based on the identified marker genes, the ceRNA network revealed an intricate regulatory interplay.

**Conclusion:**

In our investigation, we methodically identified six marker genes (AP3B2, STAT3, TSPO, S100A9, GNS, and CX3CR1). An effective diagnostic prediction model emerged from an exhaustive analysis within the training group (AUC 0.930, 95%CI 0.887–0.965) and the validation group (AUC 0.977, 95%CI 0.935-1.000).

**Supplementary Information:**

The online version contains supplementary material available at 10.1186/s12887-024-04555-y.

## Introduction

Sepsis is a medical condition characterized by an uncontrolled host response to infection, and it is presently the primary cause of mortality among critically ill patients across the globe [1]. In the United States, the current incidence rate of sepsis is approximately 3‰, with severe sepsis accounting for at least 200,000 deaths every year [2]. Neonatal sepsis, which refers to a systemic infection with bacteremia that arises within the first month of an infant’s life, is a primary cause of neonatal mortality, with its associated death rate posing a significant challenge to global health. Neonatal sepsis can be classified as early- or late-onset, with 72 h post-birth as the dividing line. Neonatal infections are responsible for about 26% of deaths in children under five years old [3]. In 2022, low- and middle-income countries reported a neonatal sepsis incidence of 17.7% and a mortality rate of 16.2% [4]. Over the past few decades, research on neonatal sepsis has focused on various types of infections and has resulted in the emergence of primary and secondary prevention strategies.

There are two classifications of cell death: accidental cell death and programmed cell death (PCD). PCD is playing a vital role in a variety of functions. Presently, twelve types of PCD have been recognized, which include Apoptosis, Pyroptosis, Ferroptosis, Autophagy, Necroptosis, Cuproptosis, Parthanatos, Entotic cell death, Netotic cell death, Lysosome-dependent cell death, Alkaliptosis, and Oxiptosis. The discovery of the Gasdermin family and the association between pyroptosis, innate immunity, and disease has expanded the research field [5]. In 2012, ferroptosis was described as a regulated form of cell death resulting from the build-up of lipid-based oxygen species through an iron-dependent process [6]. Likewise, the latest type of cell death, known as copper death, ensues from the buildup of copper in mitochondria, which triggers the aggregation of lipidated TCA cycle enzymes through direct copper binding [7].

Numerous types of programmed cell death contribute to the pathogenesis of sepsis. Lymphocyte death in sepsis is primarily mediated through apoptosis, which involves both death receptor and mitochondrial pathways and is activated by various stimuli across different lymphocyte subsets. The death of neutrophils, especially through NETosis, has been linked to the progression of multiple organ failure in septic patients [8–10]. In a recent study conducted by Abrams et al., it was reported that strong formation of NETs is primarily observed in cases of severe sepsis, and it is linked to disseminated intravascular coagulation (DIC), which can lead to adverse outcomes [11]. Pyroptosis has been shown to have a significant role in the imbalance of hemostasis and “immunothrombosis” in sepsis, participating in the regulation of transcription factor (TF) release and activity in macrophages and endothelial cells [12]. During respiratory infections, Pseudomonas aeruginosa manipulates host polyunsaturated phosphatidylethanolamine to trigger ferroptosis in bronchial epithelium, facilitating bacterial penetration [13]. Early on in the immune response, macrophages experience a surge in iron and lipid peroxidation, and ferroptosis inducers such as RSL3, salazosulfapyridine, and acetaminophen enhance macrophage bactericidal activity [14]. In sepsis, ferroptosis acts as a double-edged sword, promoting bacterial invasion and sepsis induction while also causing immune cell death and decreased immune function. Nonetheless, it can also aid immune cells in eliminating pathogens.

Utilizing bioinformatics analysis and machine learning methods aids in comprehending the fundamental processes of neonatal sepsis by examining gene expression datasets. However, there is a scarcity of in-depth functional research on PCD in neonatal sepsis. As a result, this study focused on creating a predictive model with high diagnostic accuracy employing PCD-associated genes, and aimed to identify potential therapeutic targets for neonatal sepsis management.

## Materials and methods

### Neonatal sepsis datasets and data process

**Fig. 1:**
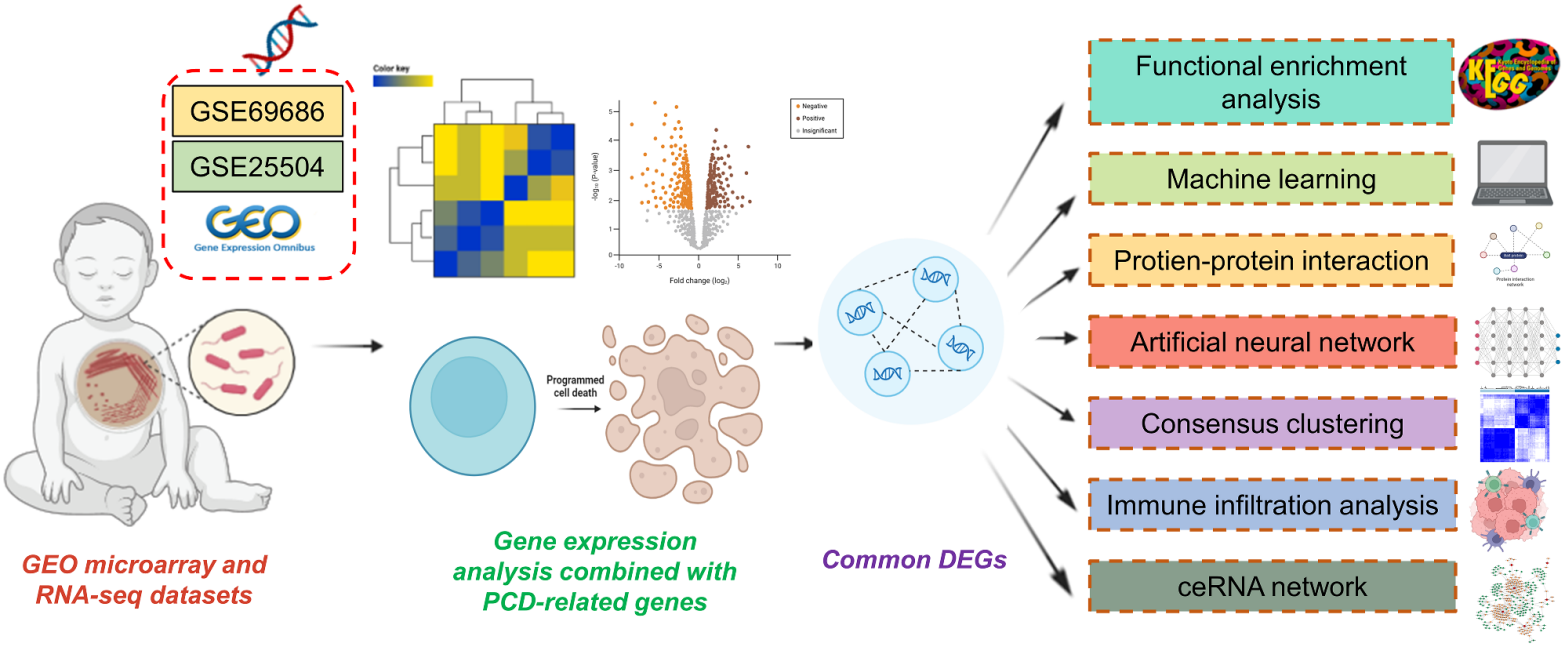
Flow chart

Gene expression data from peripheral blood samples of neonatal sepsis patients were acquired through RNA sequencing and retrieved from the Gene Expression Omnibus (GEO) database. The study involved the analysis of two distinct datasets, namely GSE69686 and GSE25504, to investigate neonatal sepsis. The diagnosis of neonatal sepsis is challenging and, to this day, neither a single over-arching definition of neonatal sepsis nor any unified diagnostic criteria exist. Positive blood culture remains the gold standard for defining newborn sepsis. We reviewed the records in the original dataset, and the inclusion criteria for neonatal sepsis are as follows: (a) persistently abnormal clinical examination (at least 2 d of clinical signs), (b) positive culture results (blood) and (c) presence of abnormal laboratory studies supporting systemic inflammation (C-reactive protein [CRP] within 48 h of evaluation > 45 mg/L). Infants with negative cultures but persistently abnormal exams and systemic inflammation were classified as having clinical sepsis. There were 64 neonatal sepsis and 85 control samples in the GSE69686 dataset, whereas the GSE25504 dataset included 170 samples from four different platforms, namely GPL570, GPL6947, GPL13667, and GPL15158. GSE69686 was selected for the primary analysis based on sample size and sequencing platforms, while GSE25504 (GPL6947 platform) was employed as the validation dataset, comprising 26 neonatal sepsis and 37 control samples. Baseline characteristics of the patients of GSE25504 (GPL6947 platform) are shown in Table S1. PCD-related genes were sourced from various locations, including the GSEA gene set, KEGG, and pertinent literature, resulting in a total of 1,257 associated genes [5, 15–17]. These genes were organized based on different types (Table S2), and the specific workflow was depicted (Fig. 1).

## Identifying DEGs between neonatal sepsis and control samples

Limma, a widely used R package, employs a generalized linear model-based methodology to detect genes with significant differences in expression, known as differentially expressed genes (DEGs). By applying the limma package (version 3.56.2) in R 4.2.1, DEGs between neonatal sepsis and control samples were determined. In this research, a P-value < 0.05 and |logFC| > 0.5 were chosen as the criteria for recognizing DEGs. Heatmaps and volcano plots of DEGs in neonatal sepsis were then generated for visualization.

### Gene function enrichment analysis

The shared genes were identified by intersecting the DEGs acquired from the analysis of neonatal sepsis and control samples with PCD-related genes. The R package “clusterProfiler” (version 4.8.3) was employed to conduct Gene Ontology (GO) enrichment and Kyoto Encyclopedia of Genes and Genomes (KEGG) pathway analyses. The statistical significance of GO terms and KEGG pathways was evaluated by considering the adjusted P-value below 0.05.

### Identification of candidate diagnostic biomarkers

To identify potential diagnostic biomarkers for neonatal sepsis, we employed three advanced machine learning techniques: the least absolute shrinkage and selection operator (LASSO) [18], support vector machine-recursive feature elimination (SVM-RFE) [19], and random forest (RF) [20]. Finally, the genes that were identified as potential gene biomarkers by all three classification models were selected for further investigation. We used a 10-fold cross-validation was employed to evaluate the models.

### Construction of protein-protein interaction networks (PPI)

The PPI was built using the user-friendly GeneMANIA website, which facilitates the generation of gene function hypotheses, the analysis of gene lists, and the determination of gene priorities when conducting functional analysis [21].

### Diagnostic model validation

ROC analysis was carried out using the pROC package (version 1.18.4) in R to determine the AUC. The signature genes’ expression was examined in both the training (GSE69686) and testing groups (GSE25504-GPL6947). Moreover, the R software’s neuralnet (version 1.44.2) was employed to create an artificial neural network using the obtained characteristic genes, establishing a highly accurate diagnostic model. Furthermore, the “ConsensusClusterPlus” package (version 1.64.0) was utilized for assessing the prediction effect.

### Nomogram model construction

In order to forecast the likelihood of neonatal sepsis, a diagnostic nomogram model was developed using the rms package (version 6.7), where the “Points” indicate the scores assigned to the relevant factor. Following that, the performance of the nomogram model in terms of prediction was evaluated through a calibration curve. Lastly, the model’s practical applicability was evaluated through decision curve analysis (DCA).

### Immune infiltration analysis

CIBERSORT is a computational technique for establishing the proportions of immune cells in neonatal sepsis and control samples to recognize varying immune cell proportions [22]. Immune cell infiltration analysis was conducted using the “Cibersort” R software package (version 1.04). Bar graphs were employed to visualize the percentage of each immune cell type across various samples. We visualized the comparison of distinct immune cell types between neonatal sepsis and control samples using vioplot. Additionally, we generated heatmaps showcasing the correlation of 22 infiltrating immune cells using the “corrplot” R package.

### GSEA

To explore the biological relevance of signature genes functionally, GSEA was executed in various subgroups [23]. KEGG gene sets were selected as the gene set database [24–26]. We used normalized enrichment score (NES) and false discovery rate (FDR) to assess the statistical significance of differences. The cut-off values for significance were set at FDR < 0.25, *P* < 0.05, and |NES| > 1.

### Construction of ceRNA network

Utilizing the starBase database, mRNA-miRNA interaction pairs were predicted [27]. Simultaneously, we obtained RNA sequences of marker genes. Using miranda software, we predicted nucleic acid binding between mRNA and miRNA. Subsequently, we utilized starBase to predict miRNA interactions and filtered the miRNA-lncRNA pairs to establish a comprehensive ceRNA network that includes mRNA-miRNA-lncRNA interactions.

### Statistical analysis

The statistical analysis was performed with the aid of R software (version 4.2.1). For comparison of groups, the Wilcoxon test was employed, with a P-value < 0.05 considered to be indicative of statistical significance. All the statistical tests we conducted were two-sided.

## Results

### Screening of DEGs in neonatal sepsis

**Fig. 2:**
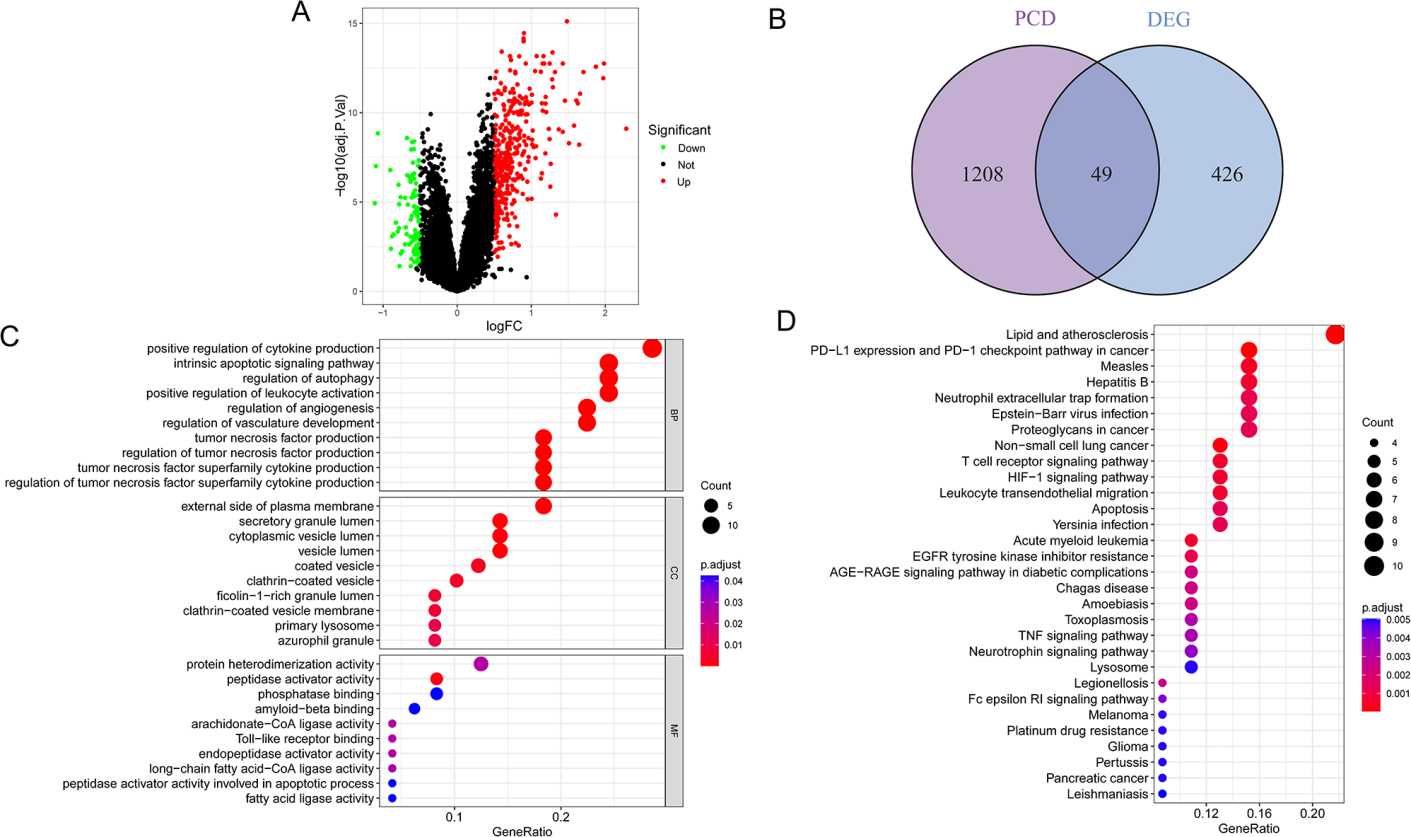
Gene expression characteristics of neonatal sepsis. (**A**) DEGs in GSE69686 between neonatal sepsis and control samples were visualized by vioplot. (**B**) Intersection of DEGs and PCD-related genes. (**C**) GO pathway enrichment analysis. (**D**) KEGG pathway enrichment analysis

We analyzed DEGs between neonatal sepsis and control samples in GSE69686 and visualized the results with a volcano plot (Fig. 2A). We detected 475 DEGs, of which 103 were upregulated and 372 were downregulated (Table S3) The heatmap displayed the top 50 dysregulated DEGs between neonatal sepsis and control samples (Figure S1). By cross-referencing the 475 DEGs with 1257 PCD-related genes, we found 49 common genes using a Venn diagram (Fig. 2B).

### Functional enrichment analysis of shared genes between DEGs and PCD-related genes

The functional enrichment analysis of the 49 common genes between DEGs and PCD-related genes was carried out. KEGG analysis showed that the main pathways enriched by these genes were “lipid and atherosclerosis,” “neutrophil extracellular trap formation,” and “T cell receptor signaling pathway” (Fig. 2C). Regarding cellular components (CC), GO analysis indicated that the shared genes were primarily located in the “external side of plasma membrane,” “secretory granule lumen,” and “cytoplasmic vesicle lumen.” The key biological processes (BP) involving these genes were “positive regulation of cytokine production,” “intrinsic apoptotic signaling pathway,” and “regulation of autophagy.” Molecular function (MF) analysis demonstrated that the shared genes were mainly involved in “protein heterodimerization activity,” “peptidase activator activity,” and “phosphatase binding” (Fig. 2D).

### Identification of potential marker genes by machine learning

**Fig. 3:**
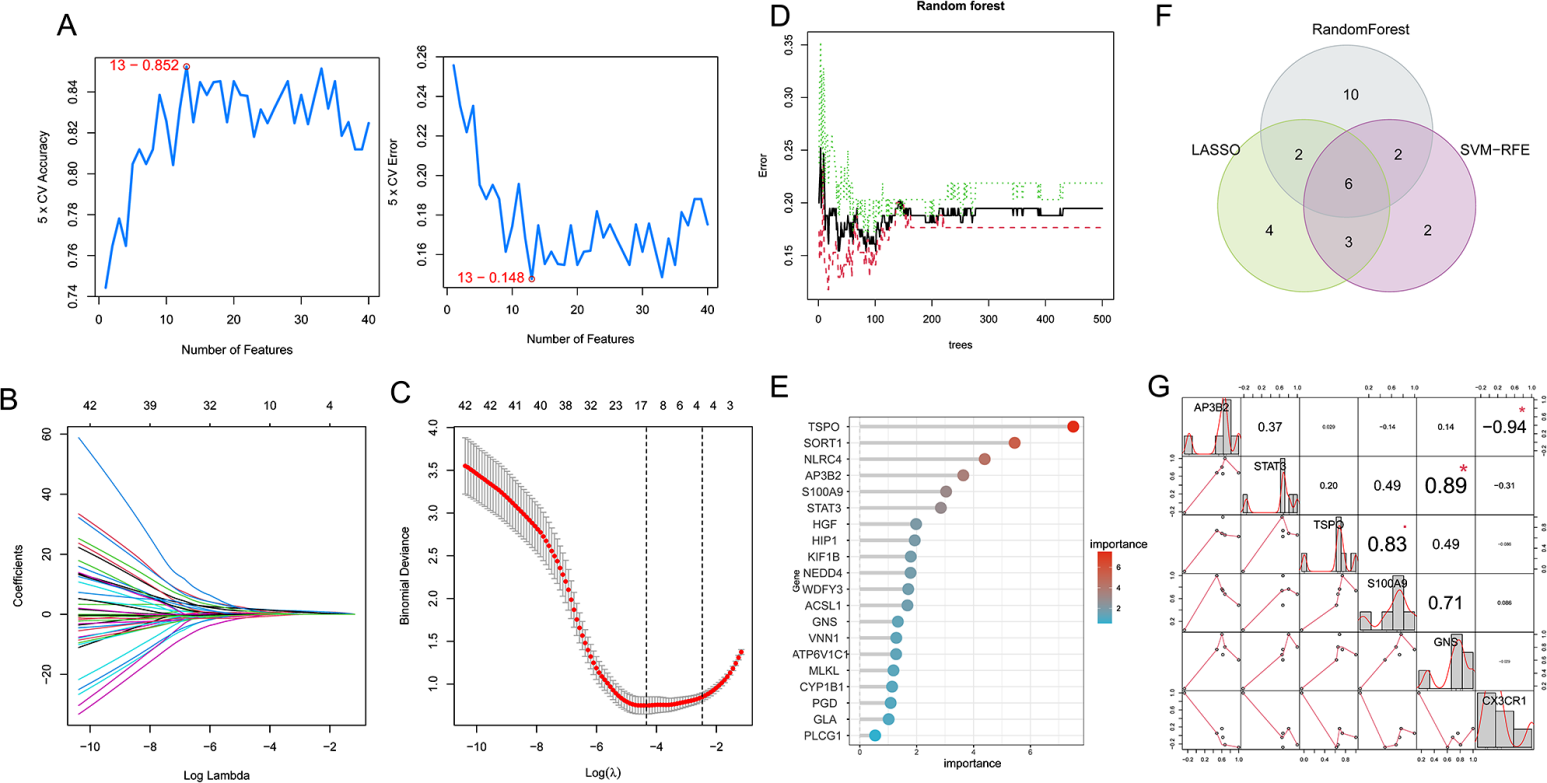
Screening potential marker genes for neonatal sepsis. (**A**) 13 diagnostic marker genes were screened by the SVM-RFE algorithm. (**B**,**C**) 15 diagnostic marker genes were screened by the LASSO regression algorithm. (**D**,**E** ) 20 diagnostic marker genes were screened by the RF algorithm. (**F**) Venn diagram of marker genes screened by LASSO, SVM-RFE and RF algorithms. (**G**) Correlation between the six maker genes in neonatal sepsis samples

To further analyze the aforementioned 49 genes, they were input into LASSO, SVM-RFE, and RF algorithms. The LASSO algorithm identified 15 genes (Fig. 3B, C), while SVM-RFE selected the top 13 genes with an accuracy of 0.852 (Fig. 3A) and an error rate of 0.148 (Fig. 3A). The RF algorithm produced 49 genes, and the top 20 in importance were chosen as the resulting genes (Fig. 3D, E). A Venn diagram was used to find the intersection of the three algorithms, and the intersecting genes were considered potential marker genes (Fig. 3F), resulting in a total of six genes (AP3B2, STAT3, TSPO, S100A9, GNS, CX3CR1). We also employed the VIF and tolerance metrics to evaluate multicollinearity. All the calculated VIF values are below the threshold of 10, and most are well below 5, further confirming the absence of significant multicollinearity among our chosen variables (Table  S4,Figure S2. GeneMANIA was employed to predict functionally similar genes for these potential marker genes. In Figure S3, the potential marker genes were positioned in the inner circle, while the predicted genes were located in the outer circle. The functional categories were primarily focused on long-chain fatty acid binding, positive regulation of DNA-binding transcription factor activity, and cellular response to interleukin-6. The chromosomal positions of the six marker genes were visualized in Figure S4. By correlation analysis, we found a strong correlation between these six marker genes (Fig. 3G).

### Validation of diagnostic model

**Fig. 4:**
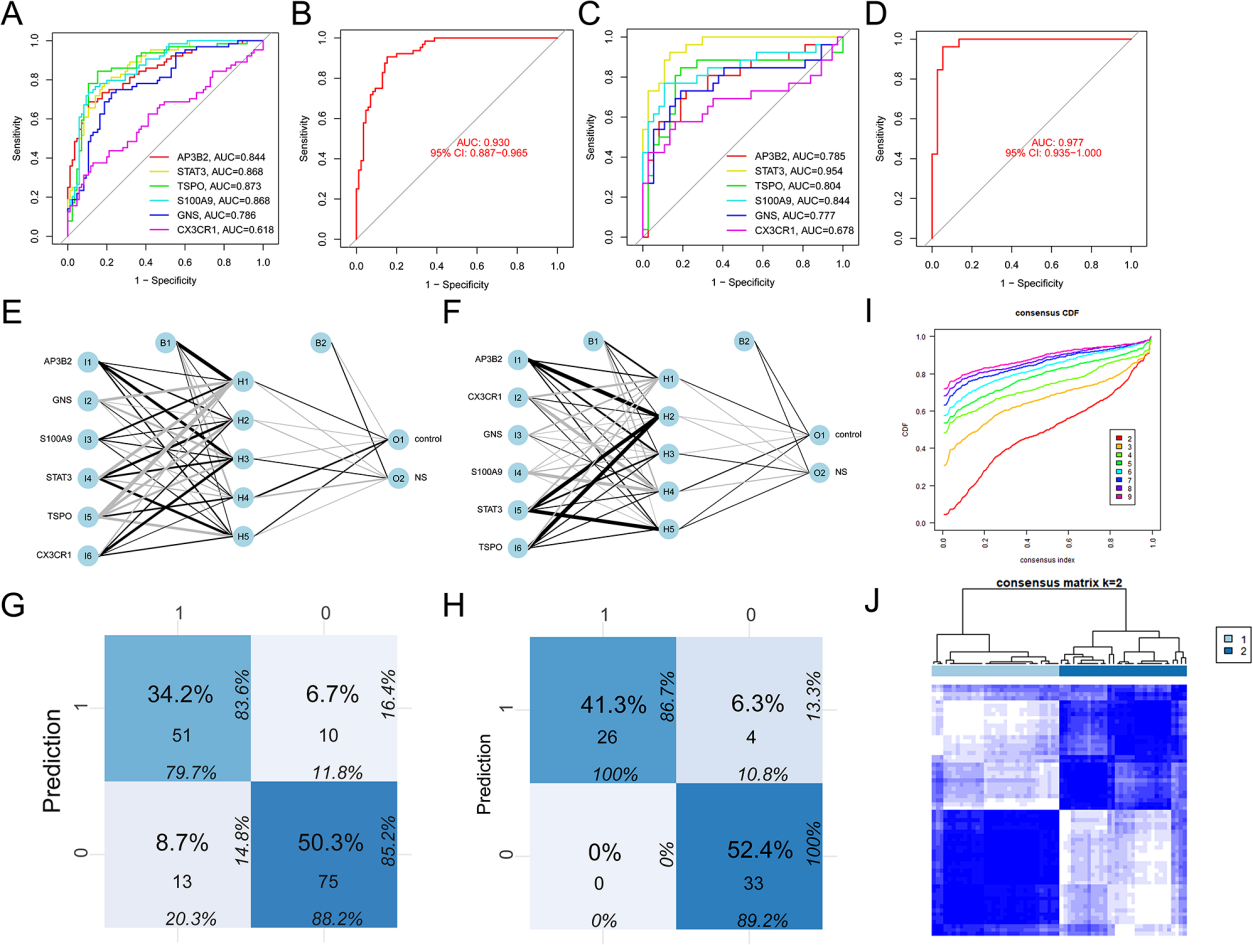
Analysis of the diagnostic value of marker genes. (**A**) ROC curve for each marker genes in training group. (**B**) ROC curve for diagnostic model in training group. (**C**) ROC curve for each marker genes in testing group. (**D**) ROC curve for diagnostic model in testing group. (**E**, **G**) Validation of the artificial neural network of the training group. (**F**,**H** ) Validation of the artificial neural network of the testing group. (**I**, **J**) consensus clustering analysis of the diagnostic model

The six potential marker genes were assessed for their diagnostic value by creating ROC curves for each gene individually and for all six genes combined, with an AUC of 0.930 (95%CI 0.887–0.965) (Fig. 4A, B). The diagnostic model was then validated in the GSE25504-GPL6947 validation group and showed excellent diagnostic significance with an AUC of 0.977 (95%CI 0.935-1.000) (Fig. 4D), with respective AUC values for each gene shown in Fig. 4C. Neural networks were also constructed using the six potential marker genes, which showed that neonatal sepsis samples could be distinguished from control samples with an accuracy of 79.7% in the training group (Fig. 4E, G) and 100% in the validation group (Fig. 4F, H). Consensus clustering analysis of the six PCD-related gene models was conducted, and the results showed that neonatal sepsis samples could be distinguished from control samples when k = 2 (Fig. 4I, J).

### A nomogram model constructed and assessed for diagnosing neonatal sepsis

**Fig. 5:**
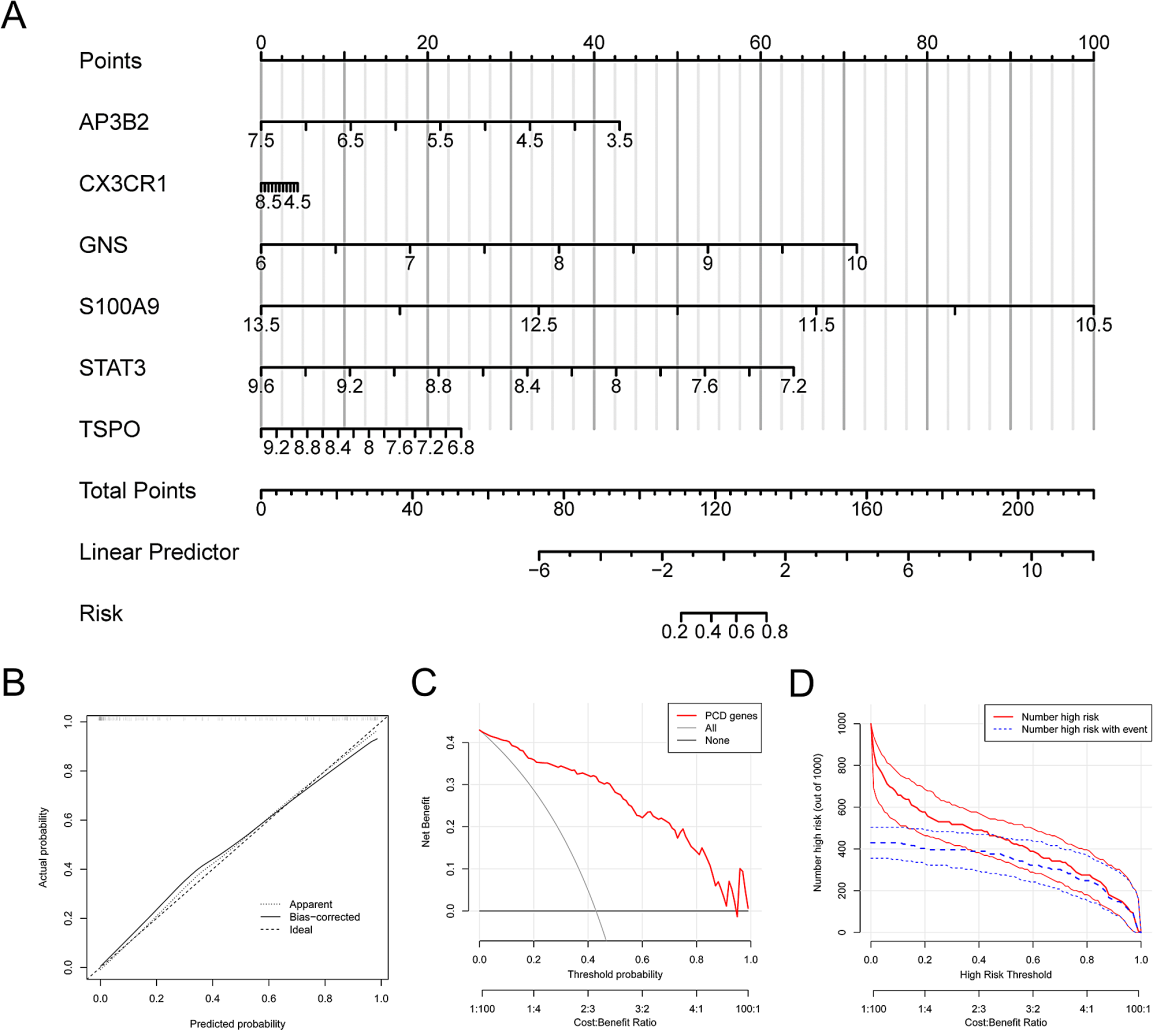
Validation and assessment of a nomogram model for neonatal sepsis diagnosis. (**A**) Nomogram model. (**B**) Calibration curve to assess the predictive value. (**C**) DCA curve to evaluate the clinical value. (**D**) Clinical impact of the nomogram model as assessed by the clinical impact curve

We employed the rms package in R to develop a nomogram model for diagnosing neonatal sepsis using six PCD-related genes (AP3B2, STAT3, TSPO, S100A9, GNS, CX3CR1) (Fig. 5A, Table S5. The model was constructed through multivariable logistic regression analysis. The nomogram model’s ability to predict the occurrence of neonatal sepsis was evaluated using a calibration curve, which revealed a negligible discrepancy between the predicted and actual incidence rates (Fig. 5B). The decision curve analysis (DCA) demonstrated the model’s potential clinical usefulness, suggesting that patients with neonatal sepsis could benefit from the model (Fig. 5C). Additionally, the clinical impact curve indicated that the model’s predictive ability was significant (Fig. 5D).

### The landscape of immune infiltration

**Fig. 6:**
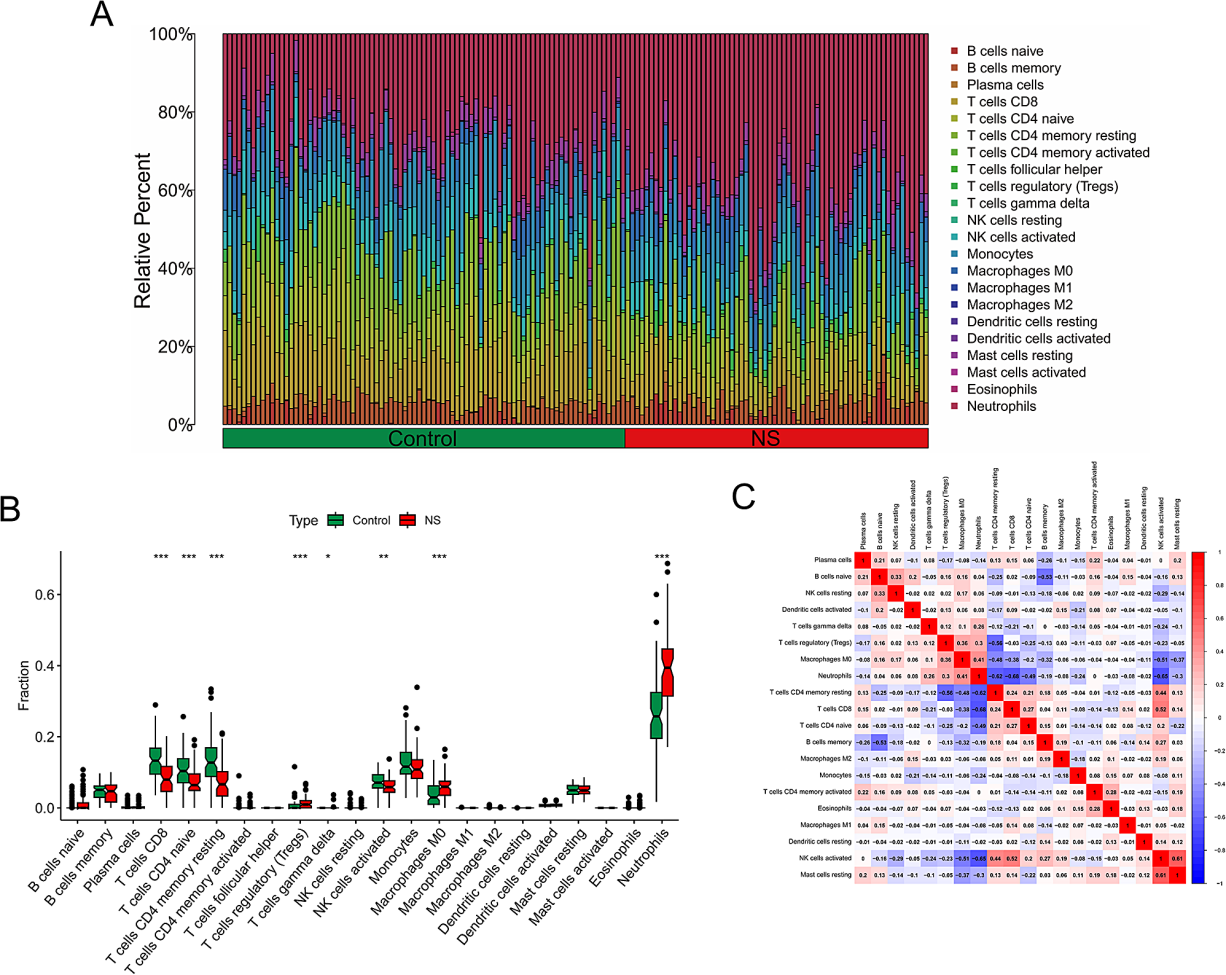
The landscape of immune infiltration between neonatal sepsis and control samples. (**A**) The boxplot diagram indicating the abundance ratio of immune cells neonatal sepsis and control samples. (**B**) The difference in immune infiltrating between neonatal sepsis and control samples. (**C**) The cor-heatmap shows the relationship between the abundance ratios of immune cells

To examine differences in immune cell composition between neonatal sepsis and control samples, we employed the CIBERSORT algorithm. The infiltration of 22 distinct immune cell subpopulations was evaluated, and the abundance ratios of these cells in the 149 samples were depicted in Fig. 6A. Additionally, the interaction of innate immune cells was illustrated in Fig. 6B. Compared to control samples, neonatal sepsis samples had a greater proportion of Tregs, macrophages M0, and neutrophils, while showing lower proportions of T cells CD8, T cells CD4 naive, T cells CD4 memory resting, and activated NK cells (Fig. 6C).

### Analysis of potential marker genes and immune infiltration

**Fig. 7:**
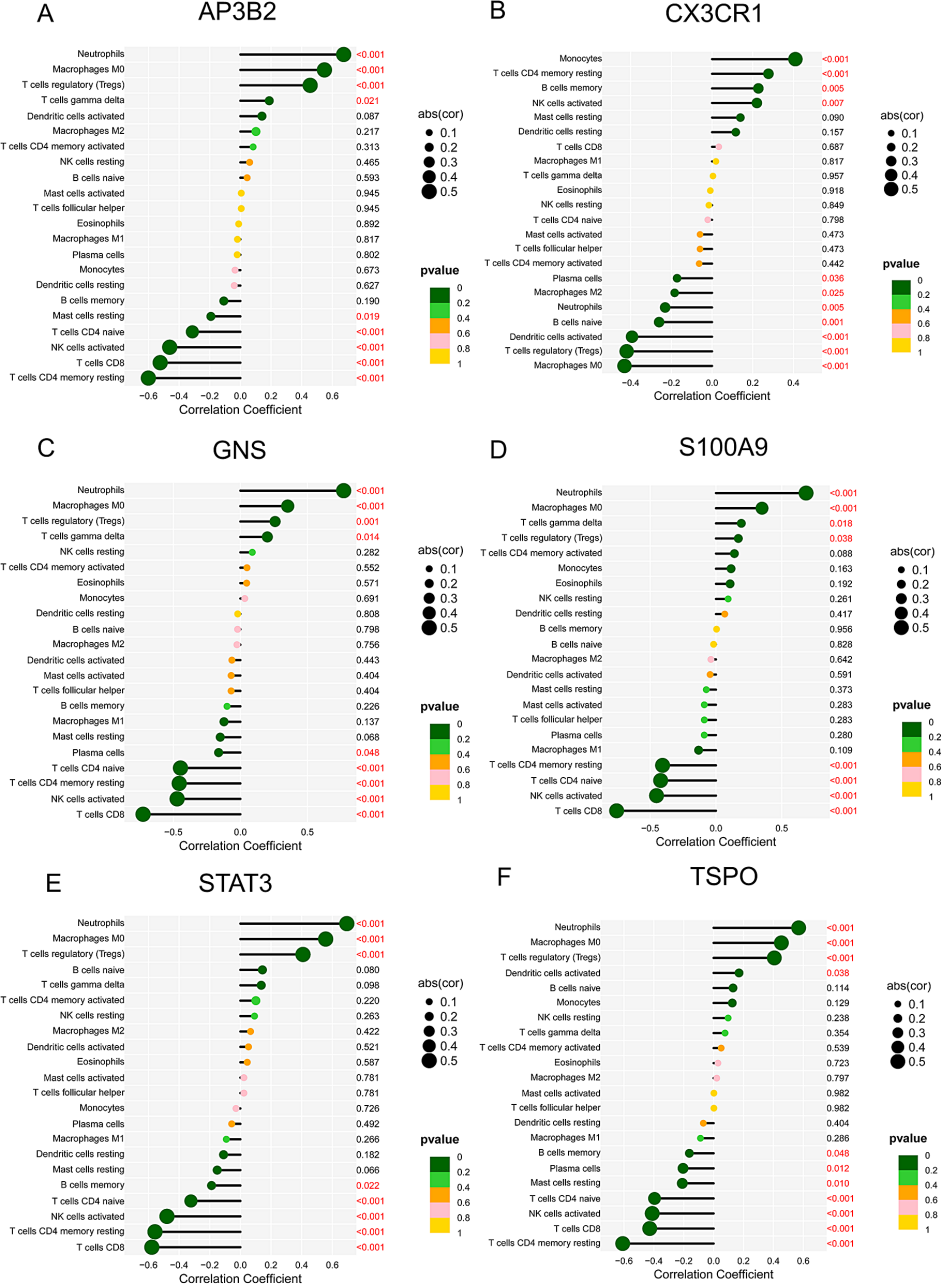
The association between the marker genes and the infiltrating immune cells level. (**A**) AP3B2. (**B**) CX3CR1. (**C**) GNS. (**D**) S100A9. (**E**) STAT3. (**F**) TSPO

Upon examining the relationship between potential marker genes and immune cells, it was observed that the expression of AP3B2, GNS, S100A9, STAT3, and TSPO was positively correlated with neutrophils, macrophage M0, and T cells regulatory (Tregs). On the other hand, CX3CR1 was found to be positively correlated with monocytes, T cells CD4 memory resting, and B cells memory (Fig. 7A-E).

### GSEA analysis of potential marker genes

**Fig. 8:**
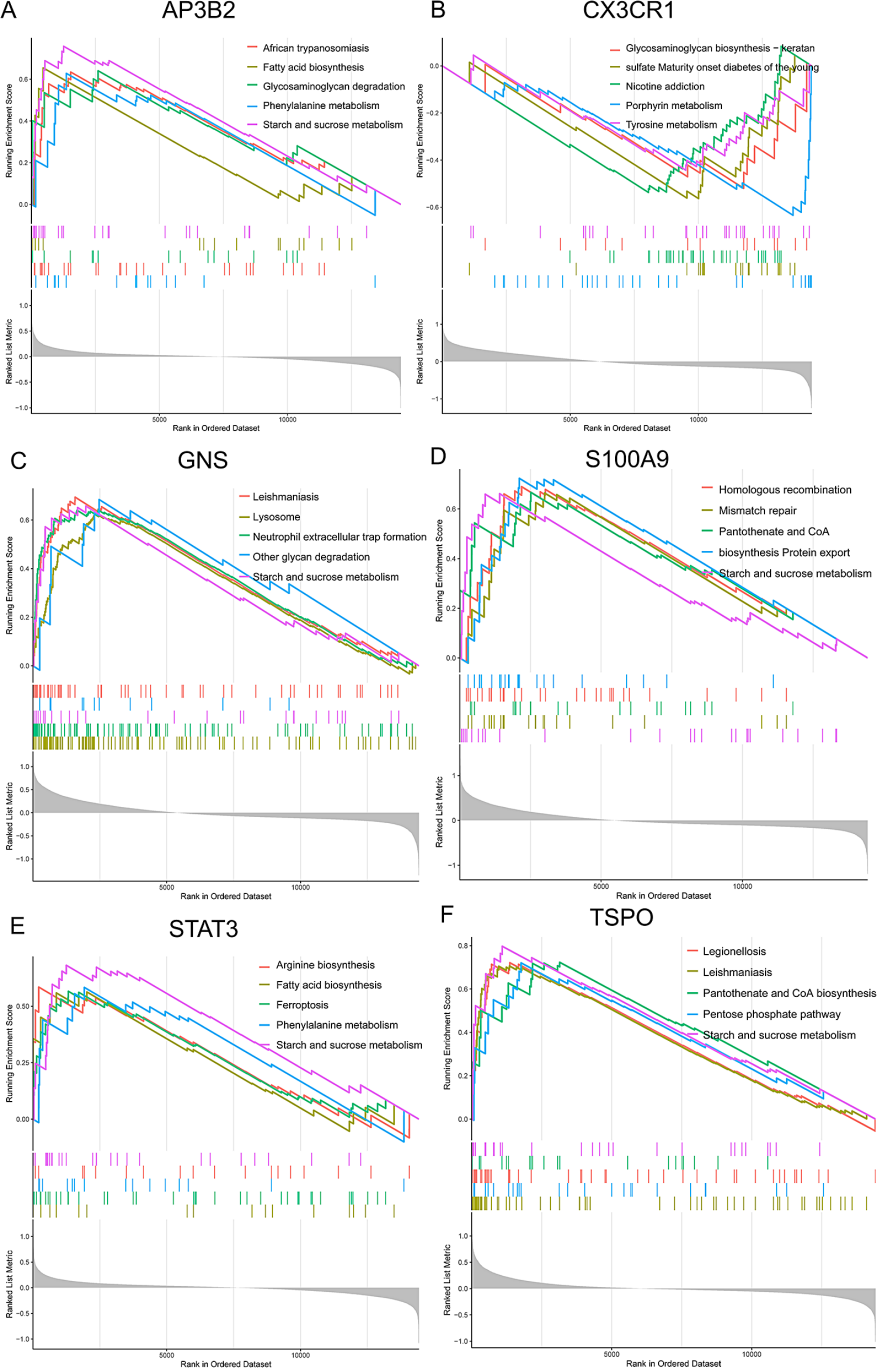
Single-gene GSEA-KEGG pathway analysis of marker genes. (**A**) AP3B2. (**B**) CX3CR1. (**C**) GNS. (**D**) S100A9. (**E**) STAT3. (**F**) TSPO

The six potential marker genes (AP3B2, STAT3, TSPO, S100A9, GNS, CX3CR1) were subjected to GSEA analysis to investigate their activities and pathways. The highly expressed genes (AP3B2, STAT3, TSPO, S100A9, and GNS) were primarily enriched in “fatty acid biosynthesis”, “glycosaminoglycan degradation”, “legionellosis”, “neutrophil extracellular trap formation”, “lysosome”, “homologous recombination”, “mismatch repair”, “ferroptosis”, and “leishmaniasis”. In addition, samples with low expression of CX3CR1 were mainly enriched in “fatty acid biosynthesis”, “maturity onset diabetes of the young”, and “mitophagy” (Fig. 8A-E). These results suggest that these genes are involved in various biological functions that may contribute to the progression of neonatal sepsis.

### A ceRNA networks based on marker genes

**Fig. 9:**
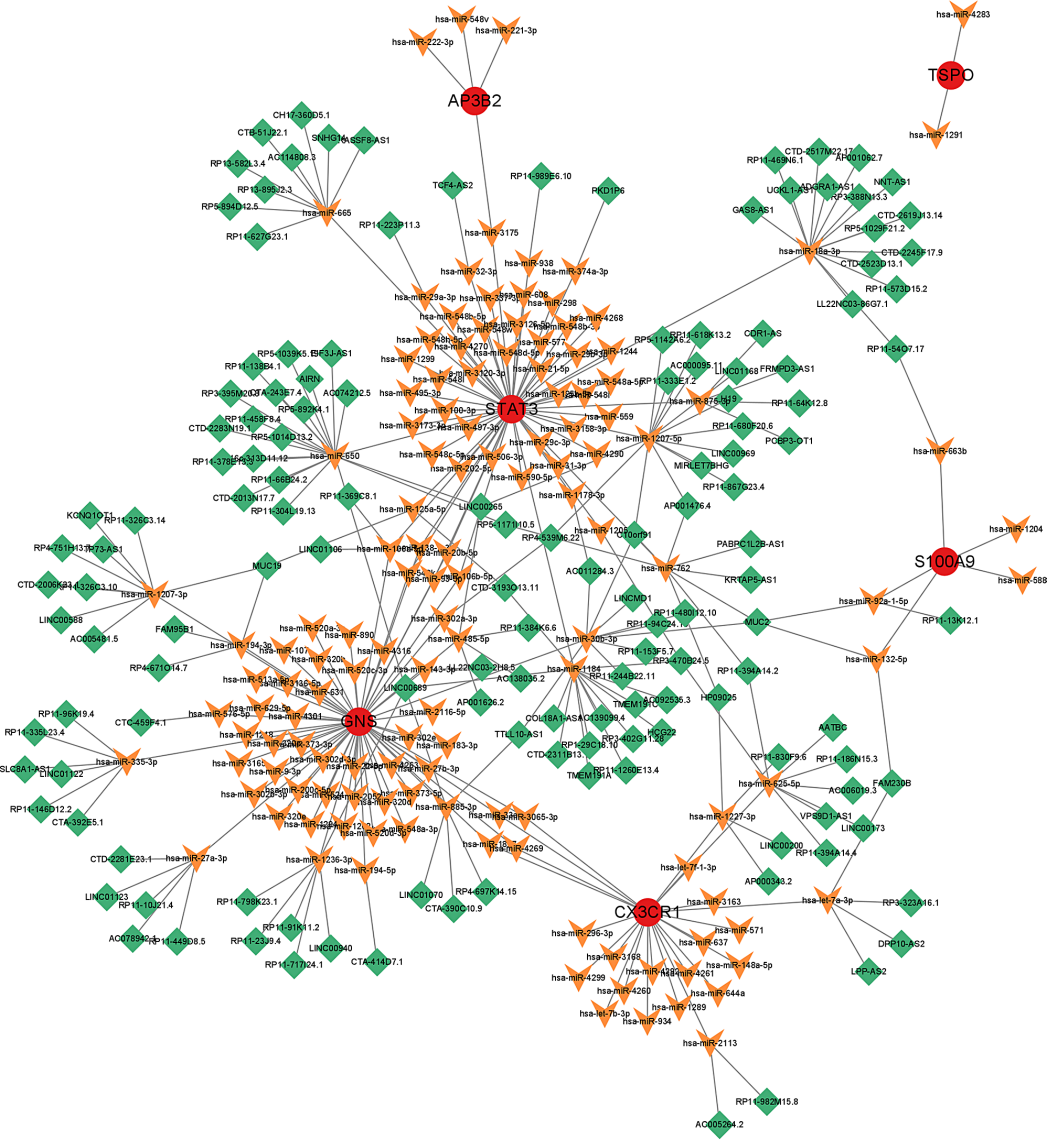
A ceRNA networks based on marker genes. The network included 311 nodes (6 marker genes, 134 miRNAs and 142 lncRNAs)

We constructed a ceRNA network by utilizing the starBase and miranda databases, which was centered on six marker genes. The network consisted of 282 nodes including 6 marker genes, 142 lncRNAs, and 134 miRNAs, with 311 edges (Fig. 9). Further analysis revealed that 18 lncRNAs could bind competitively to 4 miRNAs to regulate CX3CR1. Additionally, 50 lncRNAs could regulate the expression of GNS through competitive binding with 13 miRNAs. Moreover, in the ceRNA network of S100A9, we identified 5 lncRNAs that could combine with 4 miRNAs to regulate the gene. Lastly, 83 lncRNAs were found to be competitively bound with 14 miRNAs to affect the expression of STAT3.

## Discussion

Neonatal sepsis is a critical medical condition that can cause disruptions in microcirculation, immune function, and tissue and organ activity, leading to an increasing number of neonatal deaths worldwide. Thus, the diagnosis of neonatal sepsis and the prevention of its related complications and mortality is a major concern in global health. To address this issue, extensive research has been conducted to identify biomarkers and facilitate early intervention to minimize the risk of death. Neonatal sepsis can display an elevated inflammatory response pattern followed by an immunosuppressive phase, leading to multiple organ dysfunction. The identification of a single or group of biomarkers can be crucial in predicting, detecting and treating neonatal sepsis.

Recently, the use of machine learning techniques and public gene expression data have opened up new avenues for discovering biomarkers for disease detection. Our study focused on utilizing bioinformatics analysis and machine learning to investigate neonatal sepsis by analyzing two datasets from the GEO database. Our approach allowed us to identify different predictors compared to previous studies. Employing various algorithms to identify critical genes or biomarkers is an emerging trend, and we anticipate that it holds significant potential [28, 29]. Nevertheless, detecting prognostic indicators for disease outcomes in blood remains a challenging task, and additional laboratory and clinical investigations are necessary to validate our findings.

In this study, a set of six potential marker genes (AP3B2, STAT3, TSPO, S100A9, GNS, CX3CR1) associated with PCD were identified using LASSO, SVM-RFE, and RF algorithms. The CIBERSORT algorithm was employed to explore the immune microenvironment in neonatal sepsis and quantify the proportions of immune cells. The relationship between the marker genes and other immunomodulators was also examined. GSEA was used to identify the expression profiles of the six marker genes. Finally, a nomogram based on the six PCD-related marker genes was constructed, which demonstrated good predictive performance.

AP3B2 is a gene that encodes a subunit of the Adaptor Protein Complex 3 (AP-3) complex. The main role of the AP-3 complex is to mediate the formation of clathrin-coated vesicles in intracellular trafficking pathways and to sort and transport membrane proteins within the endosomal-lysosomal system. The AP-3 complex is composed of four subunits, including delta, beta-2, mu-3, and sigma-3 [30]. Mutations in the AP3B2 gene have been linked to various neurological disorders, such as seizures, intellectual disability, and neurodevelopmental abnormalities [31]. Recent research has focused on understanding the molecular and cellular functions of AP3B2, as well as its involvement in different cellular processes and pathological conditions. Studies have shown that AP3B2 plays a crucial role in the regulation of synaptic vesicle trafficking in neurons, which is essential for proper nervous system function [32]. Despite ongoing research on AP3B2, further investigations are necessary to understand its specific functions and involvement in different pathological conditions.

The STAT family is a cohort of transcription factors that possess the capability to bind to DNA, leading to the activation of specific signal transduction pathways and controlling gene expression. Thus, they play a vital role in cellular functions such as proliferation, differentiation, migration, maturation, and apoptosis. Recent research has emphasized the crucial role of STAT3 in sepsis pathophysiology [33, 34]. While decreasing STAT3 activity can mitigate organ inflammatory responses in LPS-induced sepsis models, conditional knockout of the STAT3 gene in macrophages and neutrophils in mice leads to an overabundance of systemic inflammation and heightened mortality rates, underscoring the critical role of STAT3 in the pathogenesis of sepsis [35].

TSPO is abundantly expressed in different tissues, including immune cells like macrophages and microglia, as well as steroidogenic tissues. TSPO is involved in several cellular processes such as cholesterol transport, steroidogenesis, porphyrin transport, regulation of mitochondrial respiration, apoptosis, and immune response modulation [36, 37]. Due to its role in immune response, TSPO has been of great interest as a potential therapeutic target in inflammatory conditions such as sepsis. During inflammation, TSPO expression is upregulated in activated immune cells, which contributes to the production of pro-inflammatory cytokines, ROS, and NO that play a significant role in the pathogenesis of sepsis [38]. Recently, studies have shown that TSPO ligands have the potential to modulate the immune response by reducing the production of pro-inflammatory mediators, promoting inflammation resolution, and exerting neuroprotective effects [39].

S100A9 belongs to a family of calcium-binding proteins known as the S100 protein family. It is encoded by the S100A9 gene and is primarily expressed in neutrophils and monocytes. S100A9 can form a heterodimer with another S100 family member, S100A8, which is also known as calgranulin A or MRP-8 [40]. The S100A8/S100A9 heterodimer, also known as calprotectin, has a variety of biological functions, including roles in inflammation, immune response, and regulation of cell proliferation and differentiation [41]. S100A9 is involved in the activation and migration of immune cells and modulation of inflammatory responses by interacting with cellular receptors such as TLR4 and RAGE [42]. S100A9 has been proposed as a potential biomarker and therapeutic target due to its involvement in sepsis. Gao et al. investigated the potential of the S100A8/S100A9 heterodimer as a biomarker for early diagnosis and prognosis of sepsis, and their results suggest that it may have clinical utility in this regard [43].

The GNS gene, which codes for the enzyme N-acetylglucosamine-6-sulfatase, plays a crucial role in lysosomal degradation of glycosaminoglycans (GAGs) like heparan sulfate and keratan sulfate. Specifically, GNS catalyzes the hydrolysis of the 6-sulfate groups of the N-acetylglucosamine residues present in these complex sugar chains. Although uncommon, abnormalities in the GNS gene can result in a rare condition known as lysosomal storage disorder. Sanfilippo syndrome type D or mucopolysaccharidosis IIID (MPS IIID), characterized by the accumulation of GAGs in cells [44]. This results in progressive neurological and systemic symptoms such as developmental delay, behavioral issues, and physical abnormalities. Although there is limited information available on the association between GNS and sepsis in the literature.

CX3CR1 is a gene that encodes the fractalkine receptor, a chemokine receptor mainly found on the surface of monocytes, macrophages, dendritic cells, certain T cells and natural killer (NK) cells. The main ligand for CX3CR1 is fractalkine (CX3CL1), a unique chemokine that exists in both soluble and membrane-bound forms. The interaction between CX3CL1 and CX3CR1 is crucial for leukocyte adhesion, activation, and migration, as well as the regulation of immune cell trafficking during both inflammation and homeostasis [45, 46]. CX3CR1 is a critical component in regulating the host immune response and the progression of organ dysfunction. Current research suggests that decreased CX3CR1 expression on circulating monocytes may represent a novel feature of immunosuppression caused by sepsis [47]. Additionally, TLR4-dependent internalization of CX3CR1 may exacerbate sepsis-induced immunoparalysis [48].

We conducted an analysis to investigate the correlation between the expression of marker genes associated with programmed cell death (PCD) and the infiltration of various immune cell types. Through GSEA analysis, we found that these marker genes were mainly involved in biological processes related to fatty acid biosynthesis, galactose metabolism, legionellosis, neutrophil extracellular trap formation, and leishmaniasis. These biological processes are believed to play a significant role in the progression of neonatal sepsis.

The marker genes identified in our study have potential applications in early diagnosis and prognostication of the disease. Early detection using these markers can aid in timely therapeutic intervention, possibly reducing the morbidity and mortality associated with late diagnosis. Furthermore, these markers can be used in tandem with existing diagnostic methods to enhance accuracy and reduce false-positive rates. The identified biomarkers can be utilized in: Rapid diagnostic tests for early detection; Stratifying patients based on the severity of the disease; Monitoring the efficacy of therapeutic interventions by tracking the expression levels of these markers. The identification of specific biomarkers for sepsis via our model can pave the way for more targeted interventions. While initial costs associated with gene expression analysis might be higher than conventional methods, the downstream benefits in terms of quicker diagnosis, precise treatment modalities, and potentially reduced hospitalization durations could be more cost-effective in the long run. Moreover, a reduction in the empirical use of broad-spectrum antibiotics could potentially mitigate antibiotic resistance, an escalating concern in modern medicine. We envision our model not as a replacement but as a complementary tool alongside blood cultures. While our model offers rapid insights, blood cultures remain invaluable for their definitive evidence of bacterial presence and for guiding antibiotic choices based on sensitivities. But there are still possible drawbacks or difficulties: (a) While the AUC values are high, it’s essential to consider the sensitivity and specificity in diverse patient populations. (b) There might be a need for specialized equipment or training to detect and quantify these markers. (c) Cost implications associated with introducing a new diagnostic tool. (d) Interpatient variability and the influence of comorbidities on the expression of these markers.

In the future work, a: One immediate strategy would be to expand model validation to datasets from various geographical regions and different demographics. This would account for genetic, lifestyle, and environmental variabilities that may impact neonatal sepsis outcomes; b: Collaborating with clinical institutions will be pivotal. Through such collaborations, we can acquire real-time patient data, which would help in refining and validating our model in near-real-world conditions. c: Exploring the integration of other relevant data types (like imaging or clinical notes) with gene expression data can enhance the model’s accuracy and robustness. d: Partnering with medical economy experts to perform in-depth cost-benefit analyses will provide a clearer understanding of the economic implications of implementing our model. e: Before full-scale clinical deployment, introducing the model to a small group of clinicians for feedback can be invaluable. This will also help in identifying any training needs for the broader medical community.

### Limitation

**Dataset limitation**: Our initial model development relied on datasets available from the GEO database. The absence of larger and more diverse datasets in this database has constrained the generalizability of our findings.

**Future validation challenges**: We aspire to validate our model using a broader clinical sample. However, the collection of such samples involves a complex, time-intensive process requiring ethical clearances, making it challenging to incorporate within the timeframe of this current study.

**Cost-effectiveness and clinical value**: We acknowledge the criticality of assessing the cost-effectiveness and clinical value of our model. A comprehensive evaluation, especially when it entails collaboration with experts in medical economics, is intricate and yet to be conducted.

**Collaborative implications**: Pursuing a comprehensive cost-effectiveness analysis and understanding the model’s true clinical value may require interdisciplinary collaborations. This could introduce additional complexities to our research.

**In vivo and in vitro validation**: Our findings also necessitate validation through both in vivo and in vitro experiments.

## Conclusions

In summary, our study has identified six PCD-related genes in neonatal sepsis and proposed a model for assessing this condition. Using bioinformatics techniques, we conducted an analysis to examine the correlation between the identified marker genes and the infiltration of immune cells. Additionally, we explored the relationships between different subpopulations of immune cells. GSEA analysis provided additional insights into the underlying mechanisms. These identified genes have the potential to serve as predictive markers and therapeutic targets for neonatal sepsis, although further research is necessary to fully elucidate the mechanisms underlying PCD-related genes and the immune microenvironment involved in neonatal sepsis.

### Electronic supplementary material

Below is the link to the electronic supplementary material.

**Supplementary Material 1: Table S1.** Baseline characteristics of the patients of GSE25504 (GPL6947 platform)

**Supplementary Material 2: Table S2.** 1257 genes related to PCD

**Supplementary Material 3: Table S3.** 475 DEGs between neonatal sepsis and control samples in GSE69686

**Supplementary Material 4: Table S4.** Table of VIF values for the 6 marker genes

**Supplementary Material 5: Table S5.** TRIPOD Checklist: Prediction Model Development and Validation

**Supplementary Material 6: Figure S1.** Heatmaps presented the expression of top 50 DEGs

**Supplementary Material 7: Figure S2.** Graphical representation of the VIF values for the 6 marker genes

**Supplementary Material 8: Figure S3.** GeneMANIA website was used to identify functionally similar genes and establish a PPI network

**Supplementary Material 9: Figure S4.** Chromosomal positions of the six marker genes

## Data Availability

The datasets (GSE69686 and GSE25504) generated during and/or analyzed during the study are available from public repository, all detailed information is recorded in Material and Methods. The original R code can be obtained at this link: https://www.jianguoyun.com/p/DXTWt5IQk4G1CRjwup4FIAA.
