# Supplementary material for: Exploration of programmed cell death-associated characteristics and immune infiltration in neonatal sepsis: new insights from bioinformatics analysis and machine learning
Source: BMC Pediatr. 2024 Jan 20;24:67. doi: 10.1186/s12887-024-04555-y (PMC10799360; doi:10.1186/s12887-024-04555-y)
Supplement: Supplementary file 1 — Supplementary Material 1: Table S1. Baseline characteristics of the patients of GSE25504 (GPL6947 platform) [file 12887_2024_4555_MOESM1_ESM.docx]

Table S1. Baseline characteristics of the patients of GSE25504 (GPL6947 platform).

| Characteristics | Control (N=37, Mean ± SD) | Sepsis (N=26, Mean ± SD) | P value |
| --- | --- | --- | --- |
| Male sex (No, %) | 23(62.16) | 14(53.84) | 0.509 |
| Corrected gestational age | 273.7 | 216.27 | <0.001 |
| Birthweight | 2993.24 | 1153.69 | <0.001 |
