# Supplementary material for: Exploration of programmed cell death-associated characteristics and immune infiltration in neonatal sepsis: new insights from bioinformatics analysis and machine learning
Source: BMC Pediatr. 2024 Jan 20;24:67. doi: 10.1186/s12887-024-04555-y (PMC10799360; doi:10.1186/s12887-024-04555-y)
Supplement: Supplementary file 4 — Supplementary Material 4: Table S4. Table of VIF values for the 6 marker genes [file 12887_2024_4555_MOESM4_ESM.docx]

**Table S4.** Table of VIF values for the 6 marker genes.

| Term | VIF | VIF_CI_low | VIF_CI_high | SE_factor | Tolerance | Tolerance_CI_low | Tolerance_CI_high |
| --- | --- | --- | --- | --- | --- | --- | --- |
| AP3B2 | 1.751488 | 1.45531 | 2.240328 | 1.323438 | 0.5709432 | 0.4463633 | 0.6871388 |
| CX3CR1 | 1.883256 | 1.551369 | 2.414916 | 1.372318 | 0.5309954 | 0.4140931 | 0.644592 |
| GNS | 5.157861 | 3.968137 | 6.824465 | 2.271092 | 0.1938788 | 0.1465316 | 0.2520074 |
| S100A9 | 2.572514 | 2.057726 | 3.337844 | 1.603906 | 0.3887249 | 0.2995946 | 0.4859732 |
| STAT3 | 2.56775 | 2.054216 | 3.33144 | 1.60242 | 0.389446 | 0.3001705 | 0.4868037 |
| TSPO | 1.706714 | 1.422786 | 2.181317 | 1.306413 | 0.5859213 | 0.4584387 | 0.7028463 |
