# Supplementary figures and images for: Exploration of programmed cell death-associated characteristics and immune infiltration in neonatal sepsis: new insights from bioinformatics analysis and machine learning
Source: BMC Pediatr. 2024 Jan 20;24:67. doi: 10.1186/s12887-024-04555-y (PMC10799360; doi:10.1186/s12887-024-04555-y)

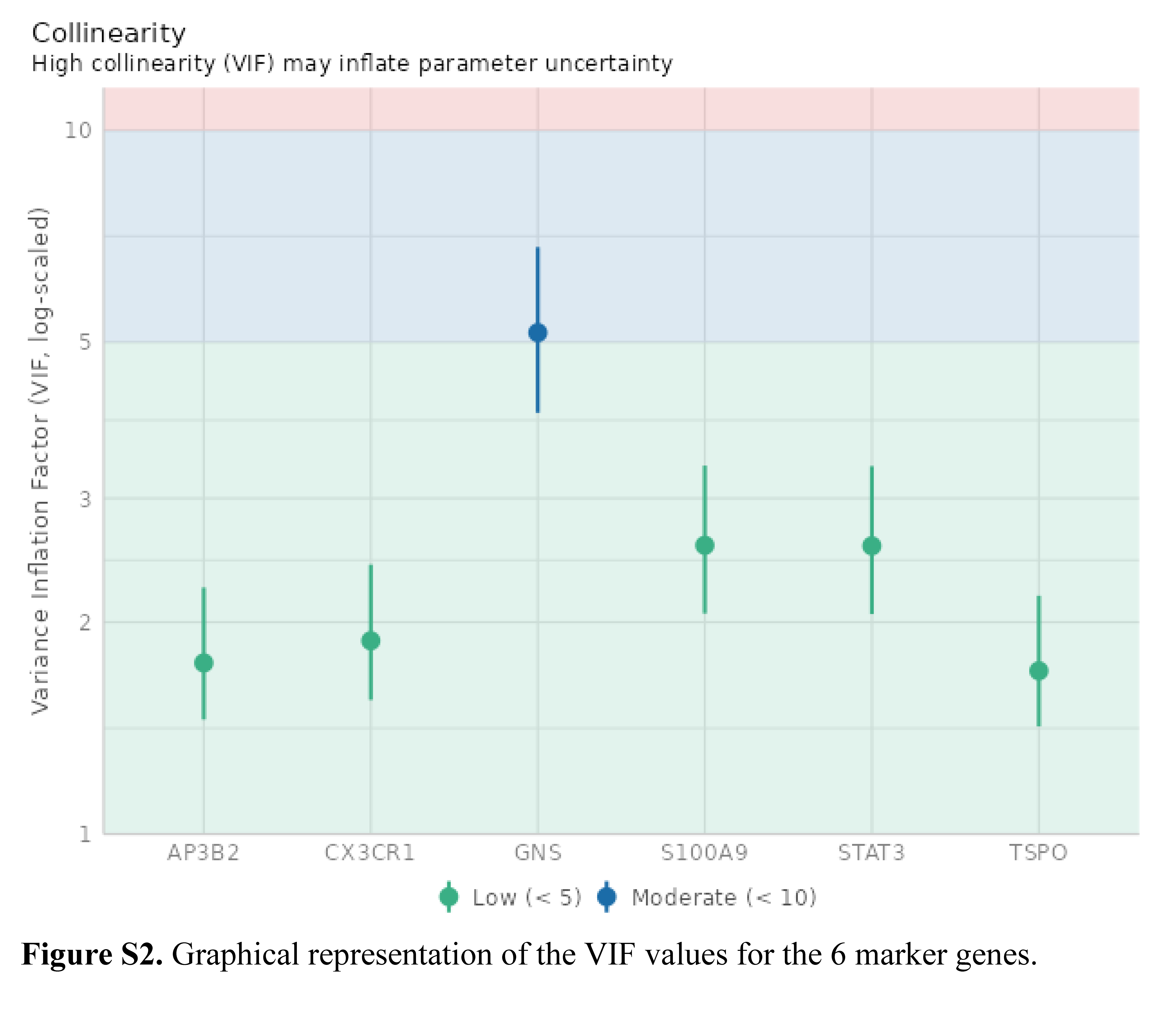

Supplement: Supplementary file 6 — Supplementary Material 6: Figure S1. Heatmaps presented the expression of top 50 DEGs [file 12887_2024_4555_MOESM6_ESM.png]

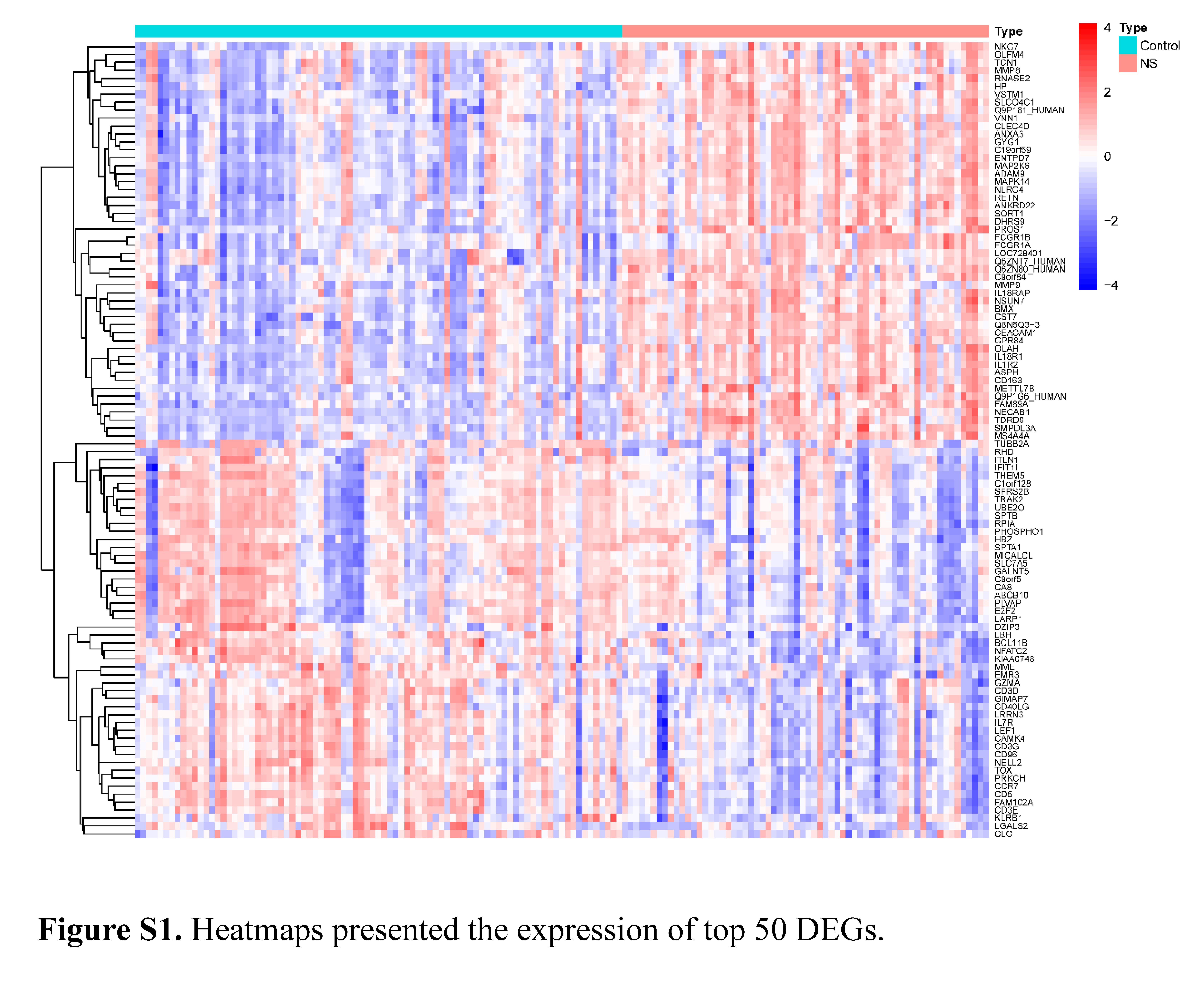

Supplement: Supplementary file 7 — Supplementary Material 7: Figure S2. Graphical representation of the VIF values for the 6 marker genes [file 12887_2024_4555_MOESM7_ESM.png]

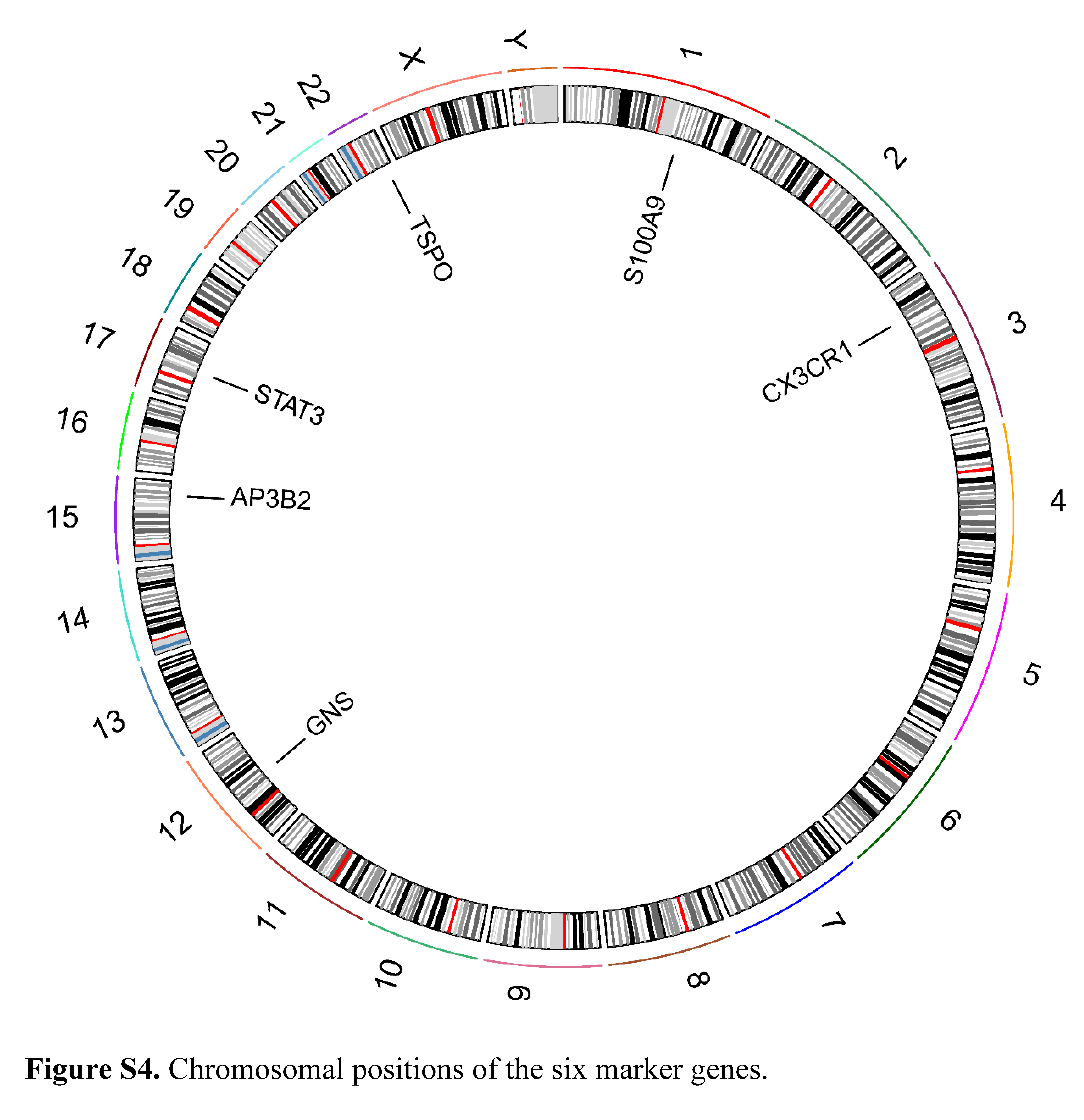

Supplement: Supplementary file 8 — Supplementary Material 8: Figure S3. GeneMANIA website was used to identify functionally similar genes and establish a PPI network [file 12887_2024_4555_MOESM8_ESM.png]

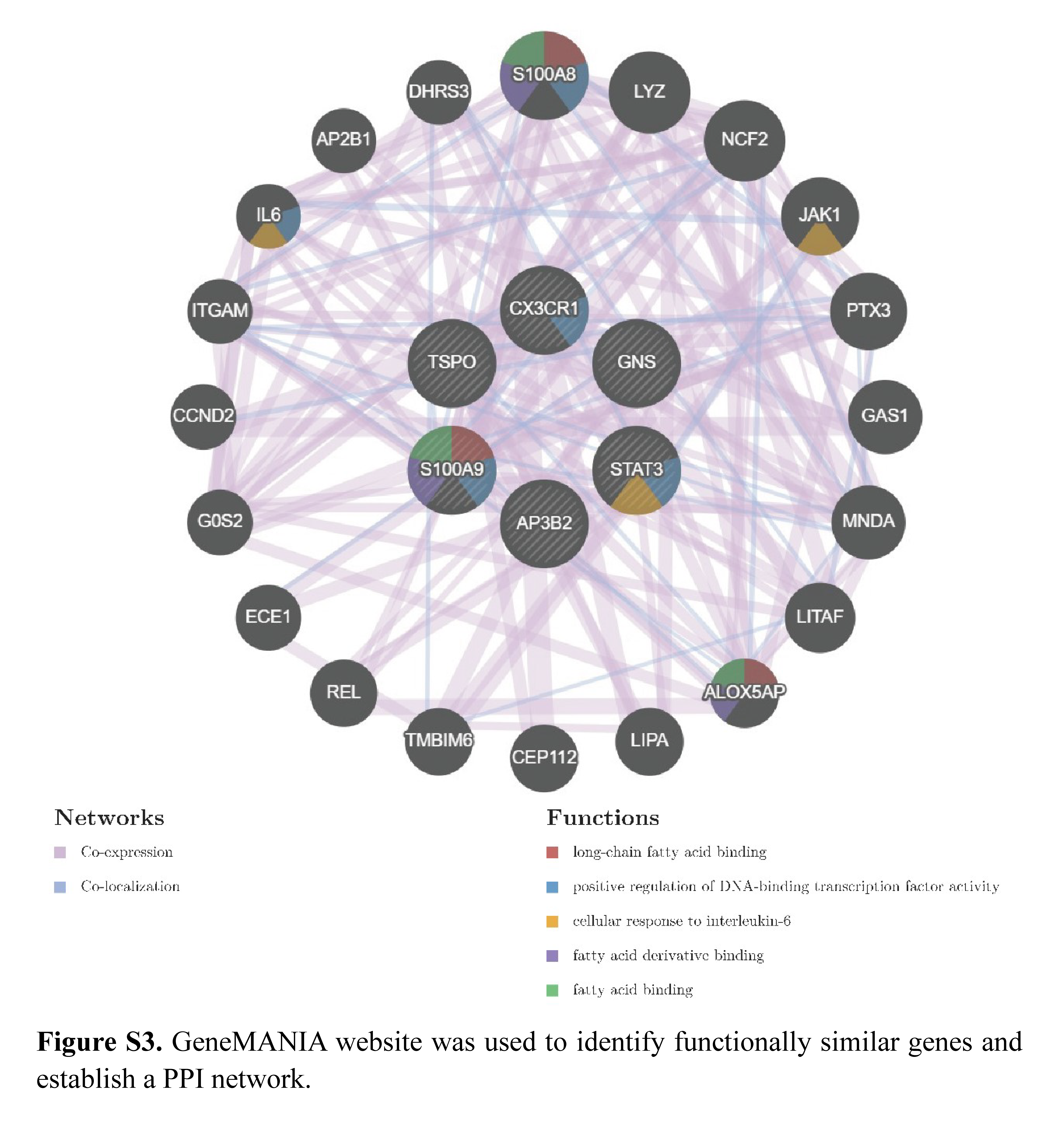

Supplement: Supplementary file 9 — Supplementary Material 9: Figure S4. Chromosomal positions of the six marker genes [file 12887_2024_4555_MOESM9_ESM.png]
